# The Effect of Beta-Boswellic Acid on the Expression of *Camk4* and *Camk2α* Genes in the PC12 Cell Line

**DOI:** 10.34172/apb.2020.053

**Published:** 2020-05-11

**Authors:** Asiyeh Jebelli, Mohammad Khalaj-Kondori, Mohammad Rahmati-Yamchi

**Affiliations:** ^1^Department of Animal Biology, Faculty of Natural Sciences, University of Tabriz, Tabriz, Iran.; ^2^Department of Clinical Biochemistry, Faculty of Medicine, Tabriz University of Medical Sciences, Tabriz, Iran.; ^3^Drug Applied Research Center, Tabriz University of Medical Sciences, Tabriz, Iran.

**Keywords:** Beta boswellic acid, *Camk2α*, *Camk4*, PC12 cells, Memory

## Abstract

***Purpose:*** Beta-boswellic acid (βBA) may play central roles in neural plasticity. Neural plasticity has significant implications for learning and memory which are governed by strict memoryrelated molecular pathways. To gain insight into the molecular mechanism by which βBA affects these pathways this study analyzed the expression patterns of *Camk2α* and *Camk4* genes in PC12 cells treated with βBA.

***Methods:*** The cytotoxic effects of different βBA concentrations on PC12 cells were examined by MTT assay. For gene expression analysis, cells were treated with concentrations of 1 and 10 µM of βBA for 12, 24, 48, and 72 hours. Total RNA was purified by RNX-Plus solution and reverse transcribed into cDNA using Thermo Scientific Reverse Transcription reagents. The expression patterns of *Camk2α* and *Camk4* genes were quantified by quantitative reverse transcription polymerase chain reaction (qRT-PCR).

***Results:*** MTT assay indicated that βBA reduced PC12 cell viability in a time- and concentrationdependent manner. The 50% inhibitory concentrations for the 48 and 72 hours time points were 35 and 26 µM, respectively; while, the βBA concentrations up to 100 µM failed to kill 50% of the cells after 24 hours. According to the qRT-PCR data, the *Camk2α* variant is not expressed in either βBA-treated or untreated PC12 cells. However, a significant upregulation was observed in*Camk4* after 12 hours of treatment with βBA, which followed by a significant downregulation after 24 hours and a persistent expression equal to the control until 72 hours.

***Conclusion:*** these findings indicate that PC12 cells not only does not express *Camk2α* but also its expression cannot be induced by βBA. However, βBA does modulate the expression of *Camk4*. This result provides further insight into the molecular mechanism by which βBA affects memory.

## Introduction

Though new and effective synthetic medicinal products have been developed around the world, interest in herbal medicines and natural therapies is increasing rapidly in industrialized countries.^1^ Within the last two decades, the dry extract of *Boswellia serrata* resin (BSE) has experienced growing popularity in animal studies as well as pilot clinical trials. The pharmacological effects of BSE are remarkably attributed to boswellic acids (BAs) which belong to the pentacyclic triterpenes.^2-4^ While BAs exist in α-or β-configurations, β-structural derivatives have superior efficacy over the respective α-isomers.^5^

BAs suppress the leukotriene biosynthesis in neutrophilic granulocytes via noncompetitive inhibition of 5-lipoxygenase, leading to a main decrease in the tumor necrosis factor-α level and thereby prevention of inflammation. In addition, BAs can inhibit elastase in leukocytes, resulting in inhibition of proliferation and inducing of apoptosis.^6-8^ Besides anti-inflammatory effects, BAs have shown anti-tumor properties in different cancers, including gastric, breast, cervical, prostate, liver, colon and pancreatic cancers.^9-12^ Moreover, the effect of beta-boswellic acid (βBA), one of the main BAs, in derivation of Dopaminergic-like neurons from Nurr1/GPX-1-embryonic stem (ES) cells and increasing of the secreted dopamine level in the differentiating-Nurr1/GPX-1-ES has been reported.^13^ In line with this neurodifferentiation impacts, βBA significantly enhanced neurite outgrowth, branching, and tubulin polymerization dynamics.^14^ These results may support this claim that βBA plays a role in the neural plasticity.

The multifunctional family of calcium/calmodulin-dependent protein kinases (CaMK) includes Ca^2+^-activated enzymes which phosphorylate different substrates in various cells. Two main members of this family are called CaMKII and CaMKIV encoded by *Camk2* and *Camk4* genes, respectively.^15^ Different isoforms of CaMKII are derived from distinct genes α, β, γ and δ. The α- and β-subunits are the predominant isoforms in the brain, where they construct dodecameric holoenzymes, composed of either one or both subunit types.^16,17^ CaMKIIα is a central component in the postsynaptic F-actin network and mediates learning and memory consolidation as well as long-term memory maintenance and storage.^17^ CaMKIV, a monomeric and multifunctional serine/threonine kinase, regulates several memory-related pathways by phosphorylating of main transcription factor cAMP response element-binding protein (CREB). Interference with CaMKIV signaling blocks late LTP and finally long-term memory formation.^18^

Our previous studies have shown that the aqueous extract of *B. serrata* improved learning and spatial memory formation and remarkably boosted *Bdnf* gene expression in the hippocampus of rats.^19,20^ Furthermore, this extract significantly prevented special memory impairment and decreased AlCl3-induced *App* upregulation in Alzheimer’s disease model rats.^21,22^ Moreover, reports by others support the positive effects of Boswellia gum resin on memory performance.^23-26^ Based on these observations and the effects of βBA on neuron growth reported in previous studies,^13,14^ the current research aimed to further unveil the molecular mechanism of the positive effects of βBA on memory by evaluating the expression patterns of the memory-related genes *Camk2* α and *Camk4* in PC12 cells. The PC12 cell line was derived from rat adrenal medullary pheochromocytoma. Possessing biochemical machinery similar to dopaminergic neurons, PC12 cells are generally used as a model system for neuronal cells to investigate neurodegenerative diseases as well as drug effects on these disorders.^27,28^

## Materials and Methods

### Materials and reagents

RPMI 1640 cell culture media, fetal bovine serum (FBS) and Penicillin-Streptomycin antibiotic were obtained from Gibco^®^ Life Technologies (Waltham, MA, USA). Ethanol, chloroform and isopropanol were purchased from Merck (Kenilworth, NJ, USA). RNX-Plus solution was obtained from SinaClon BioScience (Tehran, Iran). Reverse transcriptase, RNase inhibitor, dNTPs, and random hexamer primers were provided from Thermo Fisher**S**cientific (Waltham, MA, USA). TPP^®^ tissue culture flasks (filter cap), βBA (80342-5MG) and all other chemicals were purchased from Sigma (St Louis, MO, USA).

### Cell line

PC12 cell line was provided from the National Cell Bank of Iran (Pasteur Institute of Iran, Tehran, Iran) and cultured at 37°C in a humidified atmosphere of air containing 5% CO2. The cell culture media used in this study was RPMI 1640 supplemented with 10% FBS (inactivated at 56°C) and 1% penicillin-streptomycin. Because of the intrinsic sensitivity of PC12 cells to trypsin, the cells were mechanically detached from the culture flasks, using a scraper.

### MTT assay

3-(4,5-dimethylthiazol-2-yl)-2,5-diphenyltetrazolium bromide) (MTT), a yellow water-soluble tetrazolium dye, can be reduced by live cells to give a purple and insoluble product called Formosan that quantitated by an ELISA reader at 570 nm. Since absorbance is in proportion to the number of living cells in a sample, the MTT assay reflects the extent of cell proliferation. To perform MTT, PC12 cells were seeded into 96-well microplates in a density of 10^4^ cells/well. Six hours after seeding, the cells were put under serum starvation by media containing serum 4% and twenty-four hours following seeding, the cells were treated with concentrations 0.5, 1, 10, 25, 50 or 100 µM of βBA for 24, 48 or 72 hours. Untreated cells were considered as control. The microplates were placed in the incubator at 37°C with 5% CO2 for corresponding time intervals. Then, 20 μL MTT (5 mg/mL) solution was added to each well, and the cells were incubated at 37°C for 3h and 30 min. The culture media was then removed from each well, and 150 μL Dimethyl sulfoxide was added on it. To dissolve crystals entirely, the microplates were shacked thoroughly at room temperature for 15 minutes. The absorbance of each well was read by ELISA reader at a wavelength of 570 nm and the viability curves were drawn according to the absorbance. All treatments were done in triplicate and the experiment was repeated for three times. The 50% inhibitory concentration (IC50) was calculated for each time interval.

### Treatment of PC12 cell line by βBA for gene expression study

PC12 cells were seeded into 6-well plates in a density of 4×10^5^ cells/well and starved by RPMI supplemented with 4% serum, as mentioned for MTT assay. Twenty-four hours later, 1or10 µM of βBA were added into each well in triplicate format and treated for 12, 24, 48 or 72 hours. Untreated cells were used as control in each time interval. The cells were incubated at 37°C for corresponding time intervals. The experiments were repeated for three times.

### RNA extraction and Reverse transcription

Total RNA was extracted from the treated and untreated cells. Briefly, the cells were first washed by phosphate-buffered saline and detached from the plate. Then they were resuspended in RNAX plus for 5 minutes. 200 µL chloroform was added to each sample, kept on ice for 5 minutes, and centrifuged (12000 rpm for 15 minutes) at 4°C. The aqueous phase was precipitated with an equal volume of isopropanol. Finally, RNA pellet was washed with 75% ethanol and dissolved in DEPC-treated water. The quality of extracted RNA was evaluated on agarose gel and RNA quantity was measured by NanoDrop 1000 Spectrophotometer (Thermo Scientific, USA).

### Quantitative reverse transcription PCR (qRT-PCR)

Total RNA was reverse transcribed to cDNA using Thermo Scientific reagents. 1μg total RNA was treated with 1 U reverse transcriptase, 1 U RNase inhibitor, 1 mM dNTPs and 8 μM random hexamer in a total volume of 20 μL at 42°C for 60 minutes. The cDNA was used for quantification in a Corbett real-time system (Corbett Life Science, Australia). Primers were; *GAPDH* Forward: 5՜-CATAGACAAGATGGTGAAGGTCG-3՜ and Reverse: 5՜-CCGTGGGTAGAGTCATACTGG-3՜, *Camk2α* Forward: 5՜-GGAAACAAGAAGAATGATGG-3՜ and Reverse: 5՜-TAGGATTCAAAGTCTCCATTG-3՜, and *Camk4* Forward: 5՜-AAGCAGCCAACTTTGTCCAC-3՜ and Reverse: 5՜-TGTCTTGTCCTTGCCGTCTTG-3՜. PCR reactions were carried out in 20 μL final volumes containing 0.6 μL forward and reverse primers mix (10 pmol), 10 μL SYBR^®^ Green PCR Master Mix (2X), 2 μL diluted cDNA and ddH_2_O up to the final volume. All amplification reactions were run in duplicate. Thermal cycling was initiated with a first denaturation step of 95°C for 15 minutes and followed by a thermal profile as 95°C (30”), 60°C (20”), 72°C (20”) for 40 cycles. The Data was represented as relative expression using 2-^-ΔCT^ method. To better display the decimal numbers related to relative expression in the graph, the results were logarithmically represented.

### Statistical analysis

The MTT data was analyzed by Graph Pad Prism software version 6 (Graph Pad Inc., La Jolla, CA, USA). The data included was obtained from the triplicate treatments of PC12 cells and the three independent repeats of each experiment. The analysis of qRT-PCR data was performed by SPSS software version 19 (SPSS Inc., Chicago, MI, USA) using statistics independent Student’s t-test or One Way ANOVA. A *P* value of less than 0.05 was considered as significant.

## Results

### Effect of βBA on the PC12 cells viability 

**Figure 1:**
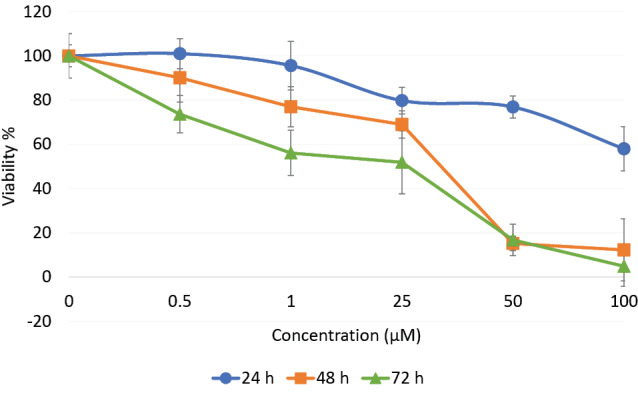
**MTT assay.** PC12 cells were seeded into 96-well microplates in a density of 10^4^ cells/well and treated with different concentrations of βBA for 24 h, 48 h and 72 h time intervals. After the time intervals, MTT was added to each well and incubated for 3 hours and 30 min. The formazan crystals were dissolved by adding DMSO and the absorbance was read by ELISA at 570 nm. Cell viabilities were plotted against βBA concentrations for the three time intervals. Data was expressed as mean ± SD from three independent experiments of triplicate treatments.

Cytotoxic effect of βBA was evaluated by MTT assay. PC12 cells were exposed to 0.5, 1, 25, 50 or 100 µM concentrations of βBA for 24, 48, or 72 hours time intervals and the cytotoxicity values were calculated. The results showed that βBA reduced the cell viability in a time- and concentration-dependent manner (Figure 1). The lower concentrations of βBA showed little cytotoxicity effects after 24 hours treatments, though the high concentration, 100 µM, resulted in 42% cytotoxicity and failed to run an IC50 for the 24 hours time interval. However, having considerable cytotoxicity values in the 48 and 72 hours time intervals, we identified βBA concentrations equal to 35 µM and 26 µM as IC50 for the 48 and 72 hours treatments, respectively.

### PC12 cell line dose not express Camk2α gene variant 

To study the expression pattern of *Camk2α* gene, PC12 cells were treated with 1 µM and 10 μM concentrations of βBA for 12, 24, 48 and 72 hours time intervals. We used these two doses for treatment of PC12 cells in the gene expression analysis experiments, based on the results obtained from our cytotoxicity assay experiments. Cytotoxicity assays introduced the 35 µM and 26 µM concentrations of βBA as IC50 values for the treatments of 48 and 72 hours time intervals, which imply that the treatments by βBA at 35 µM and 26 µM concentrations will kill 50% of the treated cells after 48 and 72 hours, respectively. Although these concentrations might be preferred for treatments with the aim of analyzing the expression of apoptosis-related genes to approve the cytotoxic effects of βBA, but due to extensive apoptosis-induced/related gene expression changes following drug treatments,^29^ they could not be appropriate doses to evaluate the gene expression patterns of other genes in somewhat physiologic conditions.

**Figure 2:**
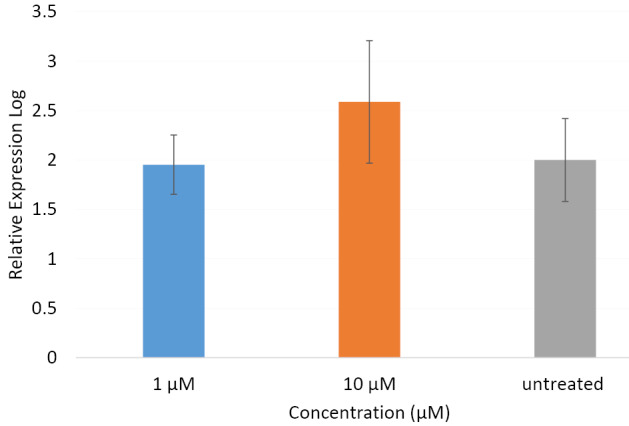
**The relative expression of** ***Camk2*** ***α*** **in B65 cells.** B65 cells were treated with 10 µM, 1 µM or 0 µM (untreated) doses of βBA and the expression of *Camk2 α* was detected by qRT-PCR. The data was normalized to *GAPDH* as an internal reference. The depicted relative expression for each concentration is the mean of three independent repeated experiments.

Results obtained from qRT-PCR revealed no expression for *Camk2α* gene both in the treated and untreated PC12 cells in the four time intervals. To warrant the specificity and functionality of the primers used for amplification of the Camk2α transcripts, we decided to test them by using RNA samples obtained from another cell line, B65, where the qRT-PCR reactions were performed in parallel for the treated and untreated PC12 and B65 cells. While no amplification was detected in samples from PC12 cells, the samples from B65 cells resulted in a considerable amplification both in the untreated and 12h-treated cells (Figure 2). We observed about equal levels of gene expressions in the samples untreated or treated with the 1 μM of βBA. The expression of Camk2α was upregulated in concentrations 10 μM of βBA. Although this change was not statistically remarkable in 12 hours time interval, treatment with βBA for other time intervals induced substantial alteration in the gene expression compared to untreated cells. These data suggest that not only PC12 cells dose not express the isoform α of the *Camk2* gene but the βBA cannot induce its expression in the PC12 cell line as well.

### βBA modulates the expression of Camk4 gene in PC12 cells

**Figure 3:**
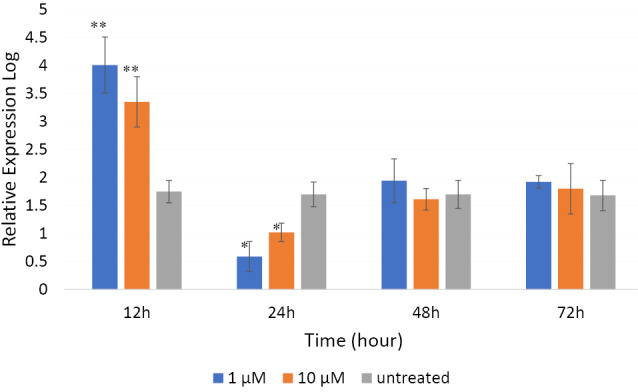
**The effect of β BA on the expression of *****Camk4*** **gene in PC12 cells.**The cells were treated with different doses of βBA for 12, 24, 48 and 72 h in triplicate format. The *Camk4* gene expression was measured by qRT-PCR and the data normalized to the *Gapdh* gene expression. Data were expressed as means ± SD from three independent experiments. ** *P* < 0.01 and * *P* < 0.05 compared to the control.

qRT-PCR was performed to reveal whether *Camk4* gene expression was affected by βBA treatment in the PC12 cells. The cells were treated with concentrations 1 or 10 μM of βBA for 12, 24, 48 and 72 hours time intervals. Untreated cells were used as control. As shown in Figure 3, both concentrations, especially the dose 1 μM, significantly increased the *Camk4* gene expression after 12 hours (*P*  < 0.01), but its expression was remarkably decreased by both doses compared to the control at the time point of 24 hours (*P*  < 0.05). This decline was more intense for the dose 1 μM than 10 μM.

In the two last time intervals 48 and 72 hours, the expression level of *Camk4* gene in the treated and untreated cells was almost similar. Although small differences were observed comparing the expression levels between the treated and untreated samples at 48 h, the differences were not statistically significant (*P*  > 0.05). A similar pattern of expression was also observed at the 72 hours time point.

## Discussion

In the current study, the PC12 cell line was treated with βBA, one of the main constitutes of Boswellia resin,^2^ and the expressions of *Camk2α* and *Camk4,* the two critical memory-related genes,^30,31^ were compared between treated and untreated cells. No expression was detected for *Camk2α* in either treated or untreated PC12 cells. This observation was in line with the findings of Tashima et al, who previously reported that the PC12 cell line expressed mainly the isoforms γ and δ of the *Camk2* gene.^32^ However, as Chen and Kelly reported earlier, treatments with all-*trans* retinoic acid, an agent that a neuron-like differentiation of PC12 cells, could induce expression of isoform α in PC12 cells.^32^ The current results, however, showed that treatments with βBA could not induce expression of the isoform α of the *Camk2* gene in this cell line. This might be due to the cell/neuron-type specific function of the different transcription initiation sits of the *Camk2α* gene. Since, its promoter showed 12- to 45-fold more activity in the neuroblastoma cell line NB2a compared with PC12, following transfection with a vector carrying the *Camk2α* promoter region.^33^ Consistently, using the neuroblastoma cell line B65, considerable expression of the isoform α was detected in both βBA-treated and untreated cells. These results further corroborate the finding that the isoform α of *Camk2* gene neither expresses nor can be induced by βBA in the PC12 cell line.

**Figure 4:**
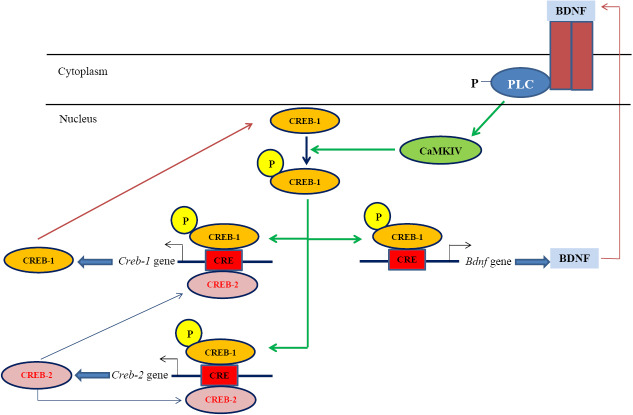
**A putative molecular mechanism by which β BA affects memory performance.** In BDNF-CaMKIV-CREB-BDNF loop, binding of BDNF to TrkB receptor triggers activation of PLC, CaMKIV and CREB. Phosphorylated and activated CREB activates the transcription of genes involved in memory consolidation including *Bdnf* gene. *Bdnf* expression leads to BDNF production which follows by binding of BDNF to its receptor and triggering of another cycle of PLC, CaMKIV and CREB activation.

Analyzing *Camk4* gene expression in PC12 cells revealed an astonishing expression pattern (Figure 3). After 12 hours, a substantial increase was seen in the βBA-treated PC12 cells compared with the untreated controls. A significant decline was observed after 24 hours, which was followed by a rise up to the *Camk4* expression level in untreated cells after 48 hours, which then approximately remained unchanged until 72 hours. This pattern of expression might indicate the involvement of some negative/positive auto-regulatory mechanisms in *Camk4* gene expression (Figure 4). As Figure 4 illustrates, CaMKIV operates in a signal transduction cascade and regulates the activity of some transcription activators such as CRE-binding protein 1 (CREB1)^34^; on the other hand, its function is controlled by other upstream regulatory proteins.^13^ It directly phosphorylates CREB1, a critical transcription factor that is involved in many aspects of the nervous system function, such as synaptic plasticity and consolidation of certain forms of memory. Phosphorylated-CREB1- forming homodimers/heterodimers bind to the conserved AMP-response elements and regulate the expression of target genes in the hippocampus.^27,30^ One of the important target genes of phosphorylated-CREB1is the brain-derived neurotrophic factor (*Bdnf* ). Binding of phosphorylated-CREB1 to the *Bdnf* promoter leads to expression and production of BDNF.^35,36^ These BDNF molecules release to the synaptic cleft where they bind to the cell-surface receptors, mainly to the tropomyosin-related kinase B (TrkB),^36^ and promote TrkB dimerization and autophosphorylation leading to the activation of the three downstream signaling cascades i.e. PLC/IP3/CaMKIV, Ras/MEK/ERK and PI3K/AKT cascades.^37-39^ For activation of the PLC/IP3/CaMKIV axis, autophosphorylation of TrkB at the Y816 residue allows recruitment of phospholipase C (PLC) which then activates the CaMKIV/CREB signaling pathway.^39,40^ Phosphorylation and activation of CREB1 in this pathway leads to the BDNF expression promoting another cycle of the BDNF-CaMKIV-CREB-BDNF activation loop (Figure 4).

Activation of the BDNF-CaMKIV-CREB-BDNF cycle by βBA might be supported by data from our previous studies. CREB1 was up-regulated in βBA- treated B65 cells after 12 and 24 hours, however, the up-regulation was largely debilitated after 72 hours.^41^ Moreover, the upregulation of BDNF in the hippocampi of rats treated with the aqueous extract of Boswellia serrata has already been reported by our group, though, no significant changes in CREB expression were observed in that study.^19^ Although the results seem controversial, they support each other in principal; the expression of CREB1 and CREB2 is regulated by two positive- and negative-feedback loops where they control the expression of each other. CREB1 induces the expression of both CREB2 and CREB1, while CREB2 represses the expression of both CREB1 and CREB2^42^ until reaching an equilibrated state, indicating that after prolonged treatment periods (e.g. 72 hours; see Patapoutian and Reichardt^38^), changes in CREB1 expression might be insignificant. This pattern of expression was observed also for CaMKIV in the present study (Figure 3). All the data highlights that βBA may affect memory performance, at least partially, through the BDNF-CaMKIV-CREB-BDNF cycle.

## Conclusion

According to the results of the current study, βBA upregulates *Camk4* in a dose-dependent manner and might promote memory performance. However*, Camk2α* neither expresses in the PC12 cell line nor can be induced by βBA. These findings provide further insight into the mechanism by which βBA affects memory performance. Further clinical trials on humans should be designed to investigate the effect of βBA on memory and learning.

## Conflict of Interest

The authors have declared that no conflicts of interest exist.

## Ethical Issues

Not applicable.

## Acknowledgments

This study was supported by the Drug Applied Research Center, Tabriz University of Medical Sciences, Tabriz, Iran.
